# Thalamo-striato-cortical determinants to fatigue in multiple sclerosis

**DOI:** 10.1002/brb3.181

**Published:** 2013-10-11

**Authors:** Maria Engström, Gullvi Flensner, Anne-Marie Landtblom, Anna-Christina Ek, Thomas Karlsson

**Affiliations:** 1 Radiology, Department of Medical and Health Sciences, Linköping UniversityLinköping, Sweden; 2 Center for Medical Image Science and Visualization (CMIV), Linköping UniversityLinköping, Sweden; 3 Nursing Science, Department of Medical and Health Sciences, Linköping UniversityLinköping, Sweden; 4 Department of Nursing, Health and Culture, University WestTrollhättan, Sweden; 5 Neuroscience, Department of Clinical and Experimental Medicine (IKE), Department of Neurology, Linköping University, County Council of ÖstergötlandLinköping, Sweden; 6 Department of Behavioural Science and Learning, Linköping UniversityLinköping, Sweden

**Keywords:** Basal ganglia, functional magnetic resonance imaging, parietal cortex, substantia nigra, working memory

## Abstract

**Background:**

The aim was to explore the thalamo-striato-cortical theory of central fatigue in multiple sclerosis (MS) patients with self-reported fatigue. If the theory correctly predicted fatigue based on disruptions of the thalamo-striato-cortical network, we expected altered brain activation in this network in MS participants while performing a complex cognitive task that challenged fatigue.

**Methods:**

MS participants with self-reported fatigue were examined by functional magnetic resonance imaging (fMRI) during the performance of a complex working memory task. In this task, cognitive effort was challenged by a parametric design, which modeled the cerebral responses at increasing cognitive demands. In order to explore the theory of central fatigue in MS we also analyzed the cerebral responses by adding perceived fatigue scores as covariates in the analysis and by calculating the functional connectivity between regions in the thalamo-striatocortical network. The main findings were that MS participants elicited altered brain responses in the thalamo-striato-cortical network, and that brain activation in the left posterior parietal cortex and the right substantia nigra was positively correlated to perceived fatigue ratings. MS participants had stronger cortical-to-cortical and subcortical-to-subcortical connections, whereas they had weaker cortical-to-subcortical connections.

**Conclusions:**

The findings of the present study indicate that the thalamo-striato-cortical network is involved in the pathophysiology of fatigue in MS, and provide support for the theory of central fatigue. However, due to the limited number of participants and the somewhat heterogeneous sample of MS participants, these results have to be regarded as tentative, though they might serve as a basis for future studies.

## Introduction

Fatigue is described as an overwhelming, abnormal feeling of extreme tiredness or exhaustion, which cannot be cured by rest or sleep. A majority of individuals with multiple sclerosis (MS) report fatigue as their most common symptom and also as their single most disabling symptom (Bakshi 2003; Krupp et al. 2010). In one study, 68% reported fatigue as either their worst or as one of their worst symptoms (Flensner et al. 2008). In a study of almost 1500 MS patients registered in the National Swedish MS-register, almost 25% of the patients without any disability and almost 50% of those with mild disability reported fatigue (Landtblom et al. 2004). Thus, fatigue is very common in MS and it also may start early in the disease course (Freal et al. 1984). Unfortunately, there is sometimes lacking interest and the knowledge about this key symptom in MS within health care is varying. Fatigue should be monitored at the clinical visits, and there are useful instruments like the Fatigue Severity Scale (FSS) (Krupp et al. 1988) and Fatigue Impact Scale (FIS) (Fisk et al. 1994a) for such purposes.

MS-related fatigue is defined as both primary and secondary fatigue. Primary fatigue is believed as caused by the disease itself, including centrally mediated processes like demyelination, axonal loss in the central nervous system (CNS), or immunological factors, as well as potential peripheral symptoms at the muscular level. Secondary fatigue is due to other influencing factors, for example, sleep problems, depression, pain, and side effects of medication (Kos et al. 2008). Central fatigue can be described as a failure to initiate and maintain both physical and mental tasks that require self-motivation in the absence of (or not related to) cognitive and motor dysfunction (Chaudhuri and Behan 2000, 2004). Despite the fact that fatigue is a common and debilitating symptom in MS, it remains a challenge. This is because no definite pathogenesis behind the symptom fatigue has been identified, although it is obvious that many factors seem to be involved. Several plausible biological backgrounds can be identified. Demyelination and axonal loss cause disruptions of the cortical and subcortical circuits that connect brain regions in functional networks (Helekar et al. 2010; Valsasina et al. 2011). This connectivity disruption can lead to cortical reorganization, and may, in several manners, reduce the efficiency of the networks. Subsequently, dysfunction of the thalamo-striato-cortical circuits may constitute a common pathophysiology for central fatigue also in other neurological disorders than MS, such as Parkinson's disease, as well as in chronic fatigue syndrome (Friedman et al. 2007). Another hypothesis of fatigue in MS underlines the potential importance of the global inflammatory processes that affect several areas in the brain; also including cortical areas (Norheim et al. 2011). This latter hypothesis is based on the frequent findings of fatigue in inflammatory and infectious diseases also where no local inflammatory foci are observed. Importantly, no consistent findings have provided unequivocal evidence for either theory, and primary fatigue in MS is probably caused by a combination of both disrupted neural circuits and pathological immune response (Kos et al. 2008).

Functional magnetic resonance imaging (fMRI) has evolved as a valuable tool for the detection of the pathophysiological mechanisms behind the symptoms in MS. The majority of previous fMRI studies report increased activation intensity and volume in MS patients compared to controls with similar performance levels (for reviews see Lenzi et al. 2008; Genova et al. 2009). Both increased activation in areas that are normally activated by a particular task, and increased bilateral activation have been observed (Lee et al. 2000; Filippi et al. 2002; Chiaravalloti et al. 2005; Sweet et al. 2006; Morgen et al. 2007).

Although fatigue is reported as one of the most debilitating symptoms of MS, only a few fMRI studies have investigated the neural determinants of fatigue. During motor tasks, increased activation in, for example, the cingulate cortex has been observed in MS patients with subjective fatigue ratings (Filippi et al. 2002) and after a mentally fatiguing task (Tartaglia et al. 2008). To our knowledge, there are only two previous studies that have monitored brain activity and fatigue in MS during cognitive tasks. DeLuca et al. (2008) found increased fatigue-related activation in frontal and parietal regions as well as in the basal ganglia and the thalamus during a modified version of the Symbol Digit Modalities Task (SDMT). Recently, Huolman et al. (2011) found increased bilateral frontal activation in MS patients with self-reported fatigue using a modified Paced Visual Serial Addition Test (mPVSAT). Similar results were found in Amann et al. (2011), although fatigue was not explicitly examined in that research.

The aim of the present study was to explore the thalamo-striato-cortical theory of central fatigue in MS patients with self-reported fatigue. If the theory correctly predicted fatigue based on disruptions of the thalamo-striato-cortical network caused by axonal loss and demyelination, we expected altered brain activation in this network in MS participants while performing a complex cognitive task that challenged fatigue. We also expected that brain activation in this network would be correlated to measures of perceived fatigue. Finally, we expected that the connections between regions of the brain that are correlated to fatigue and regions in the thalamo-striato-cortical network would be altered in MS participants.

**Figure 1:**
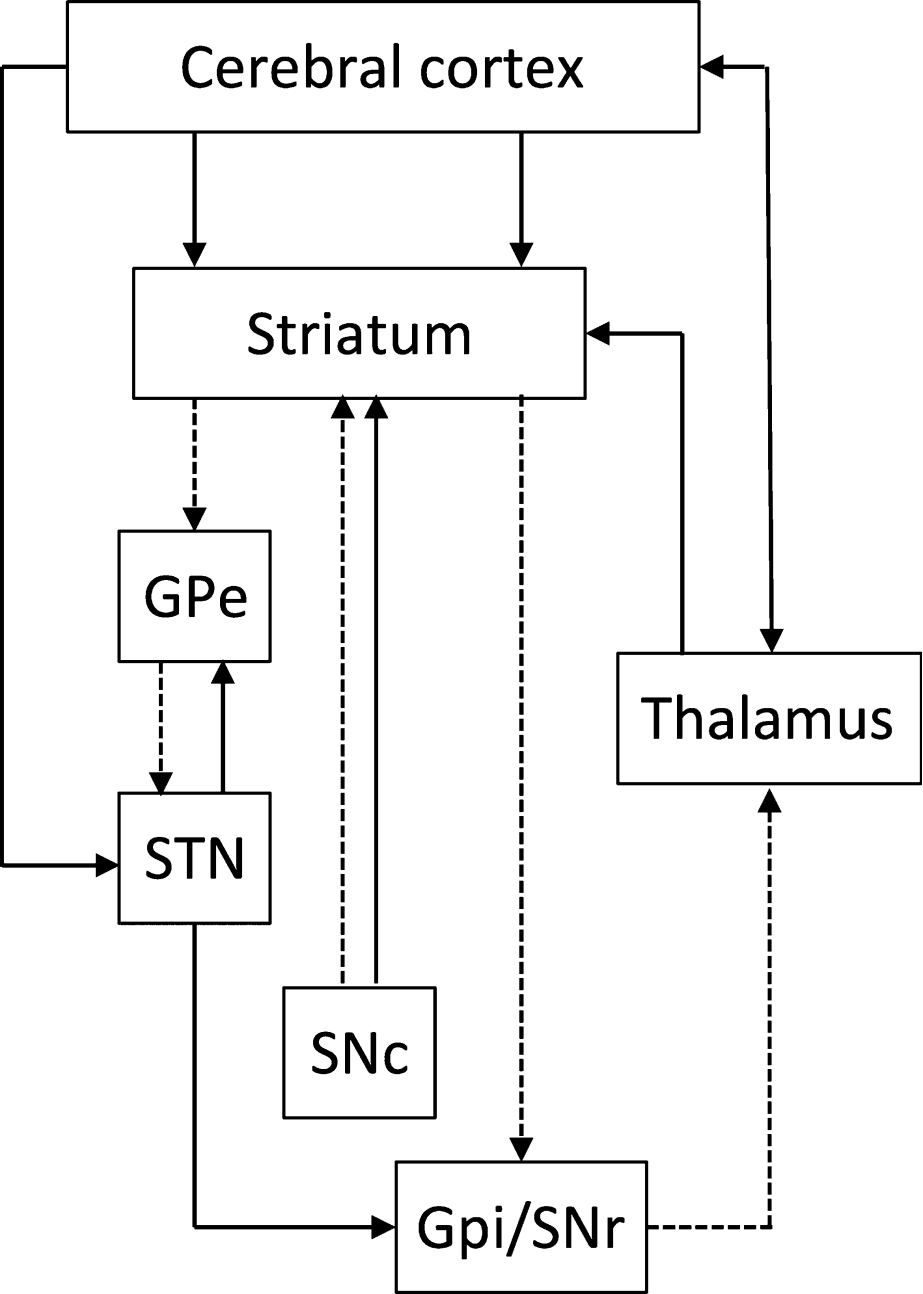
Schematic diagram of the thalamo-striato-cortical circuits that are hypothesized to be affected in central fatigue disorders. Continuous lines depict excitatory pathways and dotted lines depict inhibitory pathways. The diagram is adapted from Alexander and Crutcher (1990). GPe, globus pallidus externa; GPi, globus pallidus interna; STN, subthalamic nucleus; SNc, substantia nigra pars compacta; SNr, substantia nigra pars reticulata.

For the purpose of this study we used a complex working memory task during fMRI. This task employs a parametric design enabling analysis of the cerebral responses to increasing cognitive demands, modeled as increasing task difficulty. In this way, cognitive effort (and fatigue) is challenged. We have previously used this complex working memory task in a study of brain function in patients with periodic idiopathic hypersomnia (Engström et al. 2009, 2013). In order to explore the theory of central fatigue in MS we analyzed the blood oxygen level dependent (BOLD) responses to cerebral activity during performance of the complex working memory task by adding perceived fatigue scores as covariates in the analysis and by calculating the functional connectivity between regions in the thalamo-striato-cortical network in MS participants and controls. All image analyses were performed in regions of interest (ROIs) based on the theory of functional architecture of basal ganglia circuits described by Alexander and Crutcher (1990) (Fig. 1).

## Methods

### Participants

The present study is a continuation of a project on the topic of fatigue in MS (Flensner et al. 2008, 2011, 2013) and part of a randomized controlled trial (RCT) studying the effects of a cooling suit on fatigue, cognition, depression, and health-related quality of life. Initially individuals living in the eastern part of Sweden diagnosed with MS, registered in the Swedish MS-register and having an Expanded Disability Status Scale (EDSS) (Kurtzke 1983) in the interval 0 ≤ EDSS ≤ 6.5, age between 20 and 65 years, were invited to participate (*n* = 334), 256 responded. Among the respondents, 131 reported fatigue as one of their worst problem and heat sensitivity (Flensner et al. 2011). Power calculation indicated 48 participants, 24 in each group in the following randomized study. Exclusion criteria were ongoing use of a cooling suit and participation in another study. From 81 invited individuals, 19 responded and were included in the randomized study. Further eight participants were invited whereof six decided to participate. In all 25 individuals participated in the primary study. From this sample 16 individuals decided to participate in the fMRI study. One subject was later excluded due to excessive movement during the fMRI investigation.

Using EDSS, an individual's physical disability is measured based on ratings of neurological signs of neurological functions and ambulation, graded in twenty steps ranging from zero to ten. An EDSS = 0 indicates normal neurological conditions, while EDSS = 10 indicates the individual's death due to MS.

The mean age of the MS participants was 53.1 years (range: 42–65 years). Ten MS participants were females and five were males. The mean EDSS score was 2.8 (SD = 1.4). Descriptions of the MS participants regarding course of disease, EDSS, fatigue symptoms, and impact are found in Table 1. Of the MS patients, three received no pharmacological treatment, while 12 had different treatments. Eight were treated with immune-modulating drugs; five were treated to reduce their fatigue, and three to reduce depressive symptoms. One participant was treated with drugs to reduce forgetfulness. Two of the MS participants also had treatment toward hypertension. Two MS participants were nonnative Swedish speakers; however, they were proficient in Swedish according to a language-screening test.

**Table 1:** Descriptions of MS participants regarding course of disease and symptoms

Nr.	CD	EDSS (0–10)^1^	FF (0–5)^1^	FIS (0–160)^2^	FIS-Ph (0–40)	FIS-C (0–40)	FIS-PsS (0–80)
1	RR	4.0	4	125	36	28	61
2	PP	3.0	4	67	20	17	30
3	RR	1.0	3	32	6	10	16
4	RR	1.0	4	47	21	11	15
5	SP	6.0	5	134	40	33	61
6	RR	3.5	2	47	16	7	24
7	RR	3.0	4	89	23	24	42
8	SP	3.0	2	22	12	6	4
9	RR	1.0	5	58	16	24	18
10	SP	3.0	5	70	17	27	41
11	RR	2.5	4	73	16	15	35
12	RR	2.0	2	15	7	6	5
13	RR	4.5	5	95	28	22	45
14	RR	4.0	6	100	28	30	42
15	RR	1.0	4	28	4	10	14
Mean		2.8	3.9	66.8	19.3	18.0	30.2
SD		1.5	1.2	36.5	10.5	9.4	18.4

CD, course of disease; RR, relapsing–remitting; SP, secondary progressive; PP, primary progressive; EDSS, Expanded Disability Status Scale; FF, fatigue frequency; FIS, Fatigue Impact Scale; PH, physical; C, cognition; PsS, psychosocial.

1 Frequency of fatigue during the last 4 weeks: 0 = never; 1 = almost never; 2 = sometimes; 3 = usually; 4 = almost always; 5 = always.

2 40 items: 0 = no problem; 1 = small problem; 2 = moderate problem; 3 = large problem; 4 = very large problem; FIS physical 10 items; FIS Cognition 10 items; FIS Psychosocial 20 items.

In addition, a control group was recruited by announcement (mean age = 57.3 years, range = 47–67 years, females/males = 9/3). Imaging data from one female control were excluded due to a technical failure. Thus, imaging data are reported for 11 controls. None of the controls had symptoms of MS or fatigue according to a clinical interview. In addition, all controls spoke Swedish as their first and dominant language.

All participants were right handed according to the Edinburgh handedness inventory (scores 90–100), except one MS patient who was ambidextrous and one control who was left handed. There was no significant difference in age and years of education between MS participants and controls (*P >* 0.1). Descriptive statistics of all participants are found in Table 2. All participants gave their written informed consent to participate in the study, which was approved by the Regional Ethical Review Board in Linköping.

**Table 2:** Descriptive statistics (mean, standard deviation, *P*-value) of all participants regarding age, education (=total years of education), and cognition and results from the fatigue, depression, anxiety, and sleepiness ratings after MRI using the visual analog scale (VAS)

Measure	Controls	MS	*P*
Age	57.3 (6.4)	53.1 (6.0)	0.11
Education^1^	15.2 (4.4)	12.6 (3.4)	0.13
Vocabulary	26.5 (2.2)	23.5 (3.7)	0.03
Digit span	10.4 (2.0)	9.1 (1.8)	0.09
Working memory span	16.1 (3.3)	10.82 (3.7)	0.0002
Story recall	13.8 (4.1)	11.9 (3.9)	0.27
Complex figure test	21.2 (8.3)	12.0 (5.5)	0.009
PDQ^2^	13.6 (9.7)	24.6 (10.2)	0.013
Fatigue (VAS)	30.0 (23.6)	74.5 (27.0)	0.0002
Depression (VAS)	10.8 (8.6)	10.4 (18.5)	0.95
Anxiety (VAS)	6.6 (6.7)	7.5 (16.2)	0.86
Sleepiness (VAS)	29.0 (23.8)	58.9 (35.3)	0.023

PDQ, Perceived Deficit Questionnaire.

1 Years of education are reported for 12 MS patients.

2 Range 0–80, 20 items, 0 = never, 1 = seldom; 2 = sometimes; 3 = often; 4 = almost always.

### Procedure

On a separate occasion, 2–3 weeks before the fMRI session, the MS participants were informed about the study and interviewed using a structured questionnaire. In the questionnaire, perceived frequency of fatigue (FF) during the last 4 weeks was graded in six steps, from 0 (never) to 5 (always). To measure the impact of fatigue in daily life the Swedish version of FIS was used (Fisk et al. 1994b; Flensner et al. 2005). In FIS, 40 items covering physical (10 items), cognitive (10 items), and psychosocial dimensions (20 items) of fatigue, are graded in five steps, from 0 (indicating no problem) to 5 (indicating a major problem). The summated scores vary from 0 to 160. To estimate cognitive functions the Swedish version of the Perceived Deficit Questionnaire (PDQ; Sullivan et al. 1990) was used. In 20 items, the participant's self-reported memory, alertness, and perceived concentration functions are graded from 0 (never) to 4 (nearly always). The summated score for PDQ is 0–80. Physical disability was assessed by a neurologist using EDSS (Kurtzke 1983).

Before coming to the fMRI session, the controls were asked to fill in a health declaration form, including questions about eventual brain surgery, present medications, drug or alcohol abuse, cognitive impairments, and PDQ.

At the scanning session all participants were given a paper and pencil version of the complex working memory task (Daneman and Carpenter 1980). They were also familiarized with the task they would later conduct during fMRI scanning by performing a short version of the task on a computer. In addition, all participants carried out the Digit Span task as well as vocabulary, story recall, and complex figure tasks. Before and after the fMRI session the participants marked their actual degree of fatigue, depression, anxiety, and sleepiness on visual analog scales (VAS). The symptoms were graded from zero to ten, where zero indicated no problem and ten severe problem of each symptom, respectively.

After the scanner adjustment procedure, the participants were asked to rest with their eyes closed for 4–5 minutes while resting state fMRI images were acquired (data not reported here). Thereafter, the working memory fMRI session started, as described below.

### Complex working memory task

In the complex working memory task (working memory span) administered before the fMRI scanning, the participants listened to a set of sentences, some of which were semantically correct and some incorrect (Daneman and Carpenter 1980). The participants were instructed to report whether the sentences were correct or not and to remember the last word in each sentence. This procedure was repeated for 1–5 sentences. After the sentences had been presented, the participants were asked to recall all the target words in the correct order.

The working memory task in the scanner was similar to the paper and pencil working memory task. The main differences were (1) the sentences were presented visually for 5 sec each, (2) after each set of sentences, five words were presented for 5 sec each and the participants were asked to indicate if these words were target words or new words (lures), (3) the procedure was repeated for 1–4 sentences. The participants were instructed to answer as quickly and as accurately as they could. Task duration was 14 min. This task is described in more detail in Engström et al. (2009).

### fMRI data acquisition

Image data were acquired on a Philips Achieva (Best, the Netherlands) 1.5 T clinical scanner. For fMRI, a BOLD sensitive sequence was used (echo time (TE) = 40 msec, repetition time (TR) = 2700 msec, flip angle = 90°). Thirty-two transversal slices were acquired in interleaved fashion. The voxel size was 3 × 3 × 3 mm^3^. The number of dynamics for the complex working memory task was 302.

The complex working memory task was presented to the participants using MR compatible video goggles (Resonance Technology Inc, Northridge, CA) and Superlab software (Cedrus Corporation, San Pedro, CA). The participants made their responses using a LUMItouch button box (Photon Control Inc., Burnaby, BC).

#### fMRI analysis

Image analysis was performed using SPM8 software (Wellcome Department of Imaging Neuroscience, University College, London, UK) applying the General linear model. Images in each fMRI scan were realigned to correct for movement during scanning and normalized to the Montreal Neurological Institute (MNI) template. Thereafter, the normalized images were smoothed with an 8 mm Gaussian kernel for noise reduction and to ameliorate differences in intersubject localization.

The images were analyzed using the standard parametric design to extract brain activation with increasing task difficulty during the word recollection phase. We assumed a linear BOLD response for increasing task difficulty using a contrast vector of −3 −1 1 3, which represents each block of word recollection as separate covariates, but with different weights according to the different difficulty levels determined by the number of sentences (1–4) presented before word recollection (levels 1–4). The presentation of sentences was modeled as a separate covariate but with zero weight in the analysis.

Contrast images of each participant were used in the second level analyses. Brain activation in the control group was assessed by a one-sample *t*-test of random effects and differences in brain activation between MS participants and controls were assessed by two-sample *t*-tests. In the two-sample *t*-tests, images from one group were exclusively masked by images from the other group (mask *P*-value = 0.05, uncorrected). In this way, we obtained image maps of activation in one group that was not present in the other group.

At the whole brain level of analysis, the resulting activation maps were significance thresholded at *P* = 0.001, uncorrected. Before the ROI analysis, we used a first level threshold of *P* = 0.01. Results were reported as significant if the cluster or peak *P*-value was less than 0.05, corrected for multiple comparisons using family wise error correction (FWE).

In order to investigate if the brain activation in some of the predefined ROIs was correlated to perceived fatigue we used Fatigue VAS scores obtained after fMRI scanning as covariates in a second level analysis of all participants. In this way we obtained maps containing brain areas with activation clusters that were correlated to fatigue during the performance of the working memory task. In a subsequent test of the linear regression between the BOLD response and Fatigue VAS, the eigenvariates from the resulting correlation peaks were extracted as a measure of each participant's brain activation. The linear regression between the BOLD response and Fatigue VAS was calculated using Graph Pad Prism 5 (GraphPad Software, Inc., La Jolla, CA).

#### Regions of interest

For the purpose of this study, we created bilateral ROIs in the DLPFC and PPC to represent cortical regions that are activated by working memory and other executive tasks (Cabeza and Nyberg 2000). In addition, we created ROIs in the thalamus and the basal ganglia to represent important nodes in the thalamo-striato-cortical pathways as described by Alexander and Crutcher (1990). In Figure 1, their model of basal ganglia circuits is schematically visualized. All ROIs were created using the Wake Forest University School of Medicine (WFU) PickAtlas tool (Maldjian et al. 2003). The ROI in DLPFC was built from the lateral part of the Brodmann area (BA) 9, which was dilated by a factor of 2 in order to adjust the created ROI to the smoothed activation maps. The inferior parietal lobe, as defined in the WFU Automated Anatomical Labeling (AAL) atlas (Tzourio-Mazoyer et al. 2002), represented the PPC. Finally, the ROIs representing the thalamus, caudate, putamen, globus pallidus, substantia nigra, and the subthalamic nucleus were created from predefined masks in WFU PickAtlas.

#### Functional connectivity analysis

A seed-based functional connectivity analysis of the BOLD data was performed using the Conn software (Whitfield-Gabrieli and Nieto-Castanon 2012). Bilateral ROIs that were activated by the working memory task in controls were chosen as seeds to calculate the bivariate correlation between pairs of nodes in the thalamo-striato-cortical network. That is to say, image masks covering the DLPFC, PPC, thalamus, caudate, putamen, globus pallidus, and substantia nigra were defined as seeds for the correlation analysis (see Results section). A band-pass filter of 0.008–0.09 Hz was used in the analysis to exclude high-frequency physiological fluctuations and low-frequency nontask related fluctuations in the brain. The experimental conditions (sentence reading and word recognition at each level of difficulty) were explicitly modeled; however, in line with the standard fMRI analysis, we analyzed the data for functional connectivity during word recognition. Groups (MS and controls) were defined as covariates in the analysis. In order to obtain an overview of the connections between the nodes in the thalamo-striato-cortical network, we calculated the pair-wise correlations using a fixed effects analysis of the control group. Significant correlations (*P* < 0.05, corrected for multiple comparisons using the false discovery rate, FDR) were used to obtain a schematic picture of the network. Detailed analyses of the connectivity between the regions of the brain that were correlated to perceived fatigue (the right substantia nigra and the left PPC, see Results section) were performed by transforming the correlation matrices for further statistical analysis in SPM8. In SPM8, the same procedure for statistical thresholding as described in Section 2.4.1. was used.

## Results

### Behavioral data

**Figure 2:**
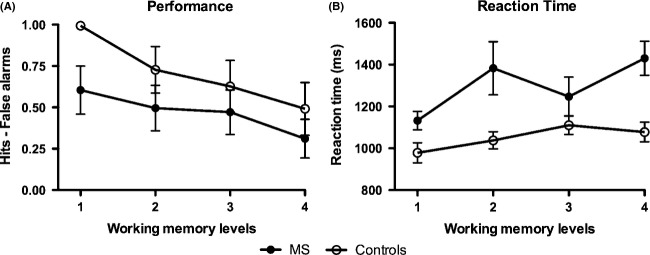
Performance during the four different difficulty levels (levels 1–4) of the working memory task administered during fMRI scanning. (A) Performance of word recognition measured as hits – false alarms. (B) Reaction time during word recognition. The lines represent mean values and the error bars represent standard error of mean (SEM).

Results from the prescanning behavioral tasks suggested a distinction between complex and noncomplex tasks. In noncomplex cognitive tasks, there were no significant performance differences between MS participants and controls. This is shown by the statistical results on the digit span (*P* = 0.09) and the story recall (*P* = 0.27) tasks, see Table 2. However, it should be noted that the differences between MS participants and controls were marginally significant for the digit span task. In complex tasks, on the other hand, the differences between the groups were highly significant. That is to say both the complex figure test (*P* = 0.009) and the working memory span task (*P* = 0.0002) resulted in significant differences between the groups. There was also a significant difference between MS participants and controls in the vocabulary task (*P* = 0.03). During the fMRI session, MS participants performed worse than controls at all levels of difficulty of the working memory task (Fig. 2A) and they also had longer reaction times (Fig. 2B). As revealed by the self-reported questionnaire (PDQ), the MS participants reported more problems with cognitive function compared to controls, *P* < 0.05 (Table 2).

After the fMRI examination, MS participants rated higher on the fatigue (*P* < 0.001) and sleepiness (*P* < 0.05) VAS compared to controls. However, there were no significant differences between the two groups in ratings of depression (*P* > 0.1) and anxiety (*P* > 0.1; see Table 2). There were no significant differences between the scorings before and after the fMRI examination in either the MS group or in the control group.

MS participants rated higher on fatigue VAS and they also performed worse on the working memory tasks administered before and during fMRI. There was a significant correlation between perceived fatigue and working memory performance during fMRI (*P* = 0.02). However, there was no correlation between fatigue scores and performance on the working memory span task administered before fMRI (*P* = 0.29).

### Brain activation in controls

**Figure 3:**
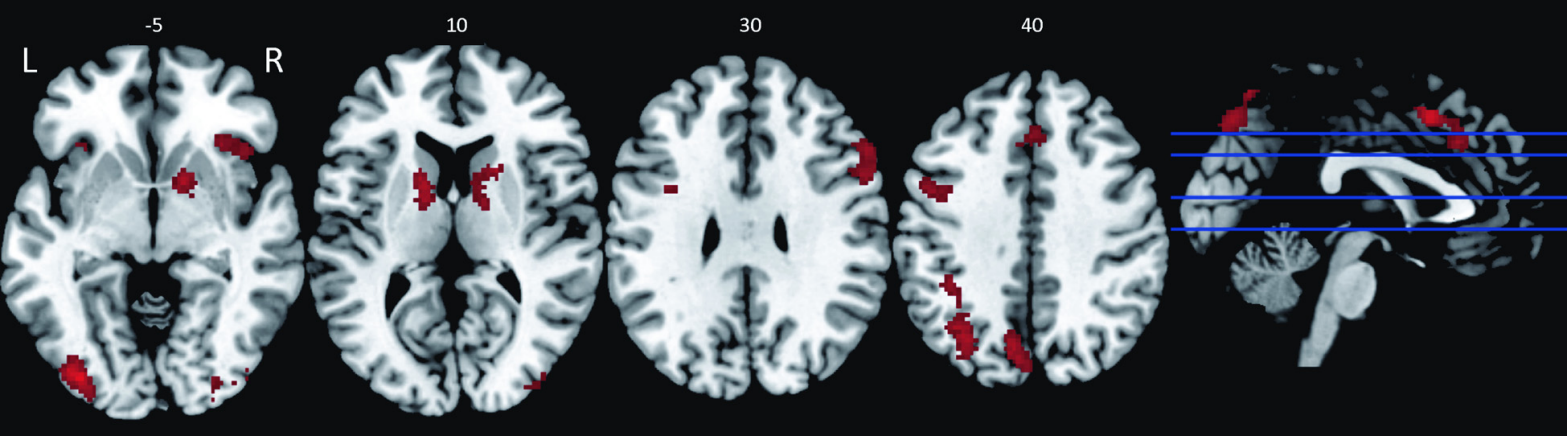
Brain activation in controls during performance of the working memory task. The figure shows significant whole brain activation at the cluster level (*P* < 0.05, family wise error [FWE] corrected for multiple comparisons) in four selected slices.

As shown in Figure 3, at the whole brain level of analysis, several cortical and subcortical areas were activated during the working memory task in controls. These results from a control group with a mean age of 57 years basically reproduce our previous results from a younger (mean age 24 years) control group (Engström et al. 2009). In the current study, significant activation (cluster *P* < 0.05, FWE corrected) was observed in the bilateral prefrontal cortex (DLPFC and the inferior frontal gyrus extending into the anterior insula) and in the left PPC. We also observed significant activation in the thalamus and striatum. Additional activation was observed in the anterior cingulate cortex, the occipital cortex, the right fusiform gyrus, and the cerebellum.

As expected, in the ROI analysis, we found significant cortical activation in the bilateral DLPFC and the left PCC at both cluster and peak levels of analysis (Table 3). Bilateral thalami were significantly activated at the cluster level of analysis. The thalamic activation clusters were particularly observed in the ventral anterior and medial dorsal parts of the thalamus. In addition, the bilateral striatum (caudate and putamen) and globus pallidus were significantly activated at the peak level. Activation in the caudate and the right globus pallidus was also significant at the cluster level. The activated areas in the caudate, putamen, and globus pallidus were merged into one cluster in each hemisphere. Finally, significant activation (peak and cluster level) was observed in the left substantia nigra. No activation was found in the subthalamic nucleus. Thus the working memory task elicited brain activation in all predefined regions of interest except the subthalamic nucleus.

**Table 3:** Brain activation in regions of interest (ROIs) during the working memory task

Region	*P*_*c*_	Size	*P*_*p*_	*x*	*y*	*z*
Controls						
Left DLPFC	<0.001	404	0.045	−46	2	40
Right DLPFC	0.005	263	0.028	58	16	28
Left PPC	<0.001	573	0.003	−48	−40	52
Left thalamus	0.015	149	0.093	−12	−10	16
Right thalamus	0.033	107	0.067	14	−8	14
Left caudate	0.013	164	0.004	−14	4	12
Right caudate	0.001	306	0.008	12	8	6
Left putamen	0.256	25	0.042	−18	6	12
Right putamen	0.168	41	0.003	18	8	−8
Left globus pallidus	0.123	19	0.035	−14	0	6
Right globus pallidus	0.038	59	0.007	14	8	−4
Left substantia nigra	0.031	25	0.002	−10	−20	−12
MS > controls						
Left PPC	<0.001	395	0.020	−42	−54	40
Right PPC	0.042	111	0.087	44	−56	42
MS < Controls						
Right DLPFC	0.115	83	0.028	58	16	28
Left thalamus	0.017	141	0.093	−12	−10	16
Left caudate	0.022	135	0.004	−16	8	12
Right caudate	0.020	180	0.008	12	8	6
Left putamen	0.256	25	0.042	−18	6	12
Right putamen	0.226	30	0.030	20	10	8
Left globus pallidus	0.165	11	0.035	−14	0	6
Left substantia nigra	0.031	25	0.002	−10	−20	−12

Family wise error (FWE) corrected *P*-value at the cluster level (*P*_*c*_), activated cluster size (size), family wise error (FWE) corrected *P*-value at the peak level (*P*_*p*_), Montreal Neurological Institute (MNI) coordinates (*x, y, z*). DLPFC, dorsolateral prefrontal cortex; PPC, posterior parietal cortex.

### Brain activation in MS

**Figure 4:**
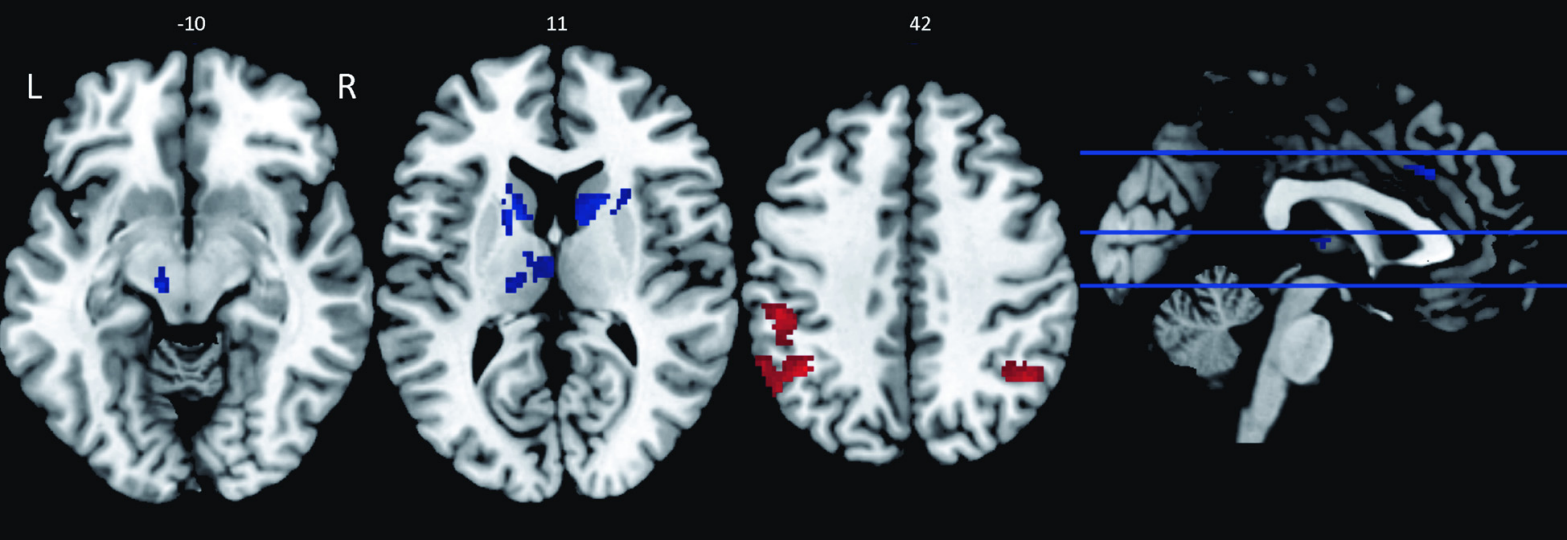
Differences in brain activation between MS participants and controls in regions of interest (ROIs). The figure shows significant differences (*P* < 0.05, family wise error [FWE] corrected for multiple comparisons) in four selected slices. The red color represents areas that were more activated in MS participants compared to controls, and the blue color represents areas that were less activated in MS participants compared to controls.

As shown in Figure 4, MS participants had more extended activation in the bilateral PPC as compared to the controls (Table 3). No other brain areas were more activated in MS participants than controls. On the other hand, MS participants had less activation than controls in almost all other ROIs, that is, the right DLPFC, the left thalamus (ventral anterior nucleus), bilateral striatum (caudate and putamen), the left globus pallidus, and the left substantia nigra. Thus MS participants activated the parietal cortex in both hemispheres more than controls, whereas they elicited less activation in the thalamus and several regions of the basal ganglia as compared to controls.

### Fatigue correlations

In order to investigate if any activation in the predefined ROIs was positively or negatively correlated to fatigue, we inserted the Fatigue VAS scores obtained during the scanning session as covariates in the analysis. The main result was that activation in the right substantia nigra was significantly correlated with fatigue (*P* = 0.02). There was also a marginally significant correlation between fatigue and activation in the left PPC (*P* = 0.08). When extracting the eigenvariate measures of the BOLD responses from the correlated activation peaks in these two ROIs we found significantly correlated activation in both regions. In the substantia nigra the correlation coefficient, *r*, was 0.69 and the *P*-value for the linear regression was less than 0.001. For the PPC the corresponding statistics were *r* = 0.77 and *P* < 0.001. Controlling for working memory performance and reaction time during the fMRI task did not significantly change these results.

**Figure 5:**
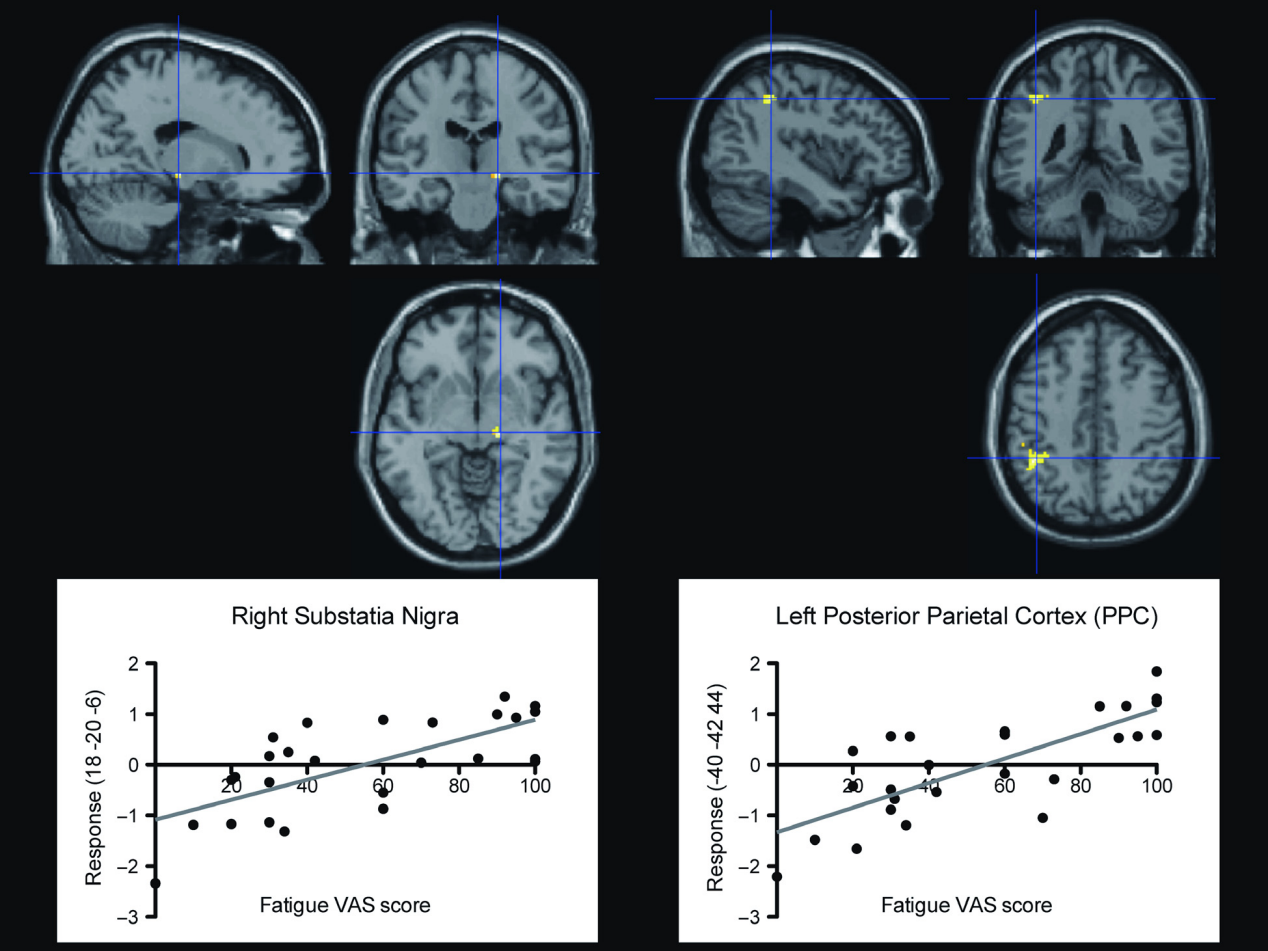
Brain activation with positive correlation to perceived fatigue during the working memory task. The images show positively correlated voxels in regions of interest: the right substantia nigra and the left posterior parietal cortex (PPC). The image of the substantia nigra was thresholded at *P* = 0.05 for visualization purpose. The image of the PPC was thresholded at *P* = 0.01, which was the threshold used in the region of interest (ROI) analysis. The graphs show the brain responses (centered around zero) at the peak of correlated activity as a function of individual visual analog scale (VAS) scores and the corresponding regression lines (gray).

The localizations of voxels in the right substantia nigra and the left PPC that were significantly or marginally significantly correlated to fatigue VAS scores and the corresponding regression graphs are shown in Figure 5. Both graphs show a positive correlation, which means that participants with higher ratings of perceived fatigue have higher activation in the right substantia nigra and the left PPC during performance of the working memory task. Note that the brain responses in Figure 5 are centered round zero, and thus the signs of the responses have no quantitative values. None of the predefined ROIs were negatively correlated to fatigue.

### Functional connectivity

**Figure 6:**
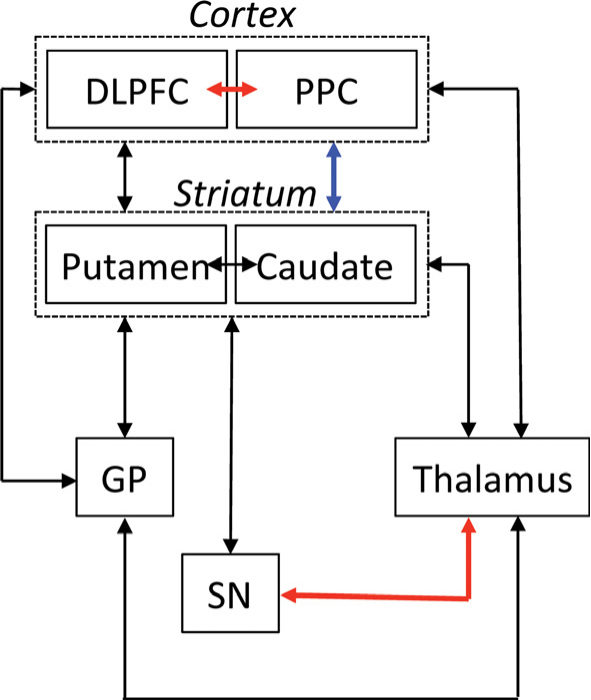
Schematic diagram of the thalamo-striato-cortical circuits, which describes the results of the present study. The red arrows describe couplings between areas that were more strongly connected in MS participants than controls during the working memory task. The blue arrow describes the coupling between areas that were more weakly connected in MS participants. DLPFC, dorsolateral prefrontal cortex; PPC, posterior parietal cortex; GP, globus pallidus; SN, substantia nigra.

The overview analysis of connections between nodes in the thalamo-striato-cortical network resulted in a schematic model (Fig. 6) that broadly resembles the theoretical model described by Alexander and Crutcher (1990) (Fig. 1). The main difference was that, due to the low anatomical detail in the predefined ROIs, we were not able to differentiate between the globus pallidus externa and interna and between the substantia nigra pars compacta and pars reticulata. Another important difference between our schematic model and the theoretical model was that we did not model the subthalamic nucleus, as this region was not activated by the working memory task. Figure 6 shows how the cortical regions (DLPFC and PPC) were connected to each other and to the striatum. The substantia nigra was coupled to both the striatum and to the thalamus. Note that in the theoretical model, the pars compacta of the substantia nigra is coupled to the striatum, whereas the pars reticulata is coupled to the thalamus. In our schematic model, the thalamus was also coupled to the cortex and the basal ganglia, as described in the theoretical model.

**Figure 7:**
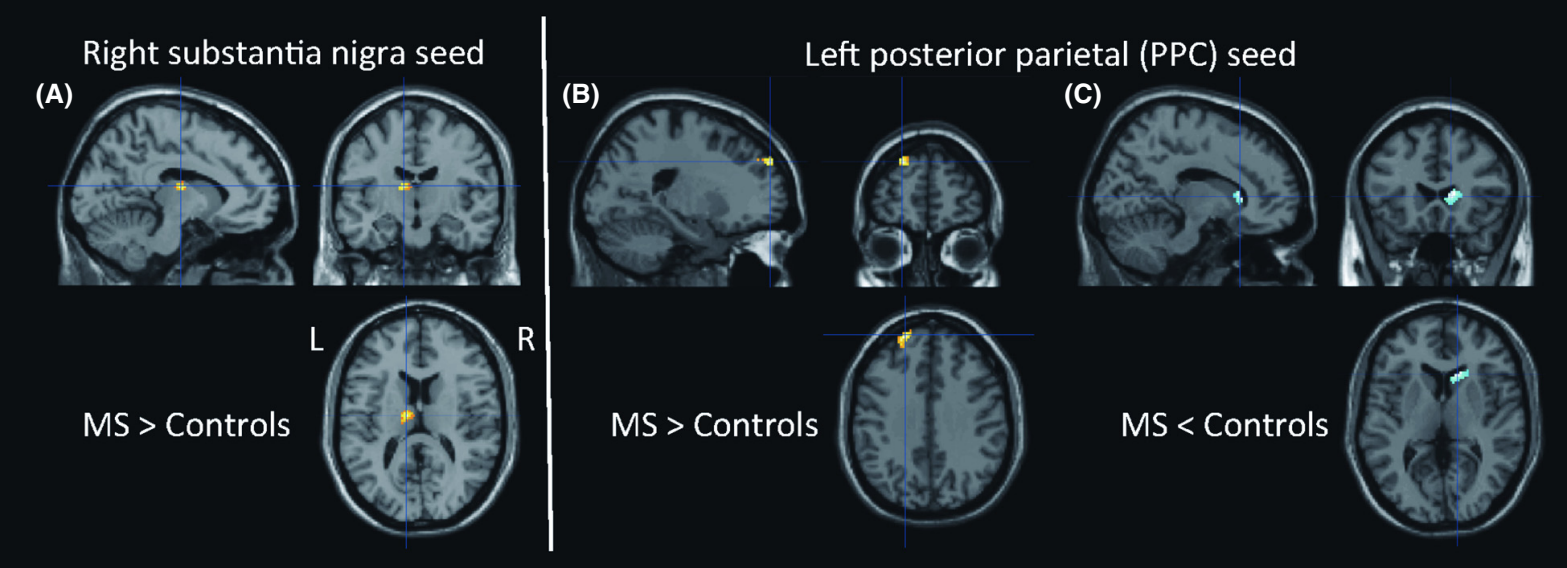
Images of regions of interest (ROIs) with different functional connectivity to the seed regions in MS participants. (A) The image shows stronger functional connectivity between the right substantia nigra and the left thalamus in MS participants compared to controls. (B) The image shows stronger functional connectivity between the left posterior parietal cortex (PPC) and the left dorsolateral prefrontal cortex (DLPFC) in MS participants compared to controls. (C) The image shows weaker functional connectivity between the left posterior parietal cortex (PPC) and the right caudate in MS participants compared to controls. L, left; R, right.

In the detailed analysis of pair-wise correlations it was revealed that MS participants had stronger couplings between the right substantia nigra and the left thalamus (−10 −12 14, *P* = 0.003). In Figure 7A, it is shown that both anterior medial and lateral aspects of the thalamus were more strongly coupled to the right substantia nigra in MS participants than in controls. The results also showed that the left PPC was more strongly coupled to anterior parts of the left DLPFC (−20 56 36, *P* = 0.012, Fig. 7B), whereas it was more weakly coupled to the right caudate head (14 22 6, *P* = 0.037, Fig. 7C) in MS participants compared to controls. The couplings with different functional connectivity in MS participants and controls are visualized in the schematic diagram of the thalamo-striato-cortical network in Figure 6. The red arrows show that MS participants had stronger couplings within the cerebral cortex (PPC ⇒ DLPFC) and within subcortical regions (Substantia nigra ⇒ Thalamus) compared to controls. The blue arrow in Figure 7 shows that MS participants had weaker couplings between the cerebral cortex and striatum (PPC ⇒ Caudate).

## Discussion

During performance of the complex working memory task, the MS participants showed increased activation in the bilateral PPC. This finding is in line with previous studies that also found increased bilateral cortical activation in MS patients, especially in regions that are normally activated by the administered task (Chiaravalloti et al. 2005; Sweet et al. 2006; Morgen et al. 2007). Frequent findings of hyperactivation in MS patients have been interpreted as a compensatory reorganization in order to maintain normal performance (Lenzi et al. 2008; Genova et al. 2009). However, the hypothesis of compensatory brain networks in MS patients is challenged by an alternative hypothesis proposed by Hillary et al. (2006) and Hillary (2008). They argue that increased brain activation in MS patients is a response to increased cognitive demand, which in turn is associated with poorer performance. This argument is well in line with the neural efficiency hypothesis, discussed by Neubauer and Fink (2009). The results in this study support the latter theory, because the MS participants performed worse than the controls during the complex working memory task, and still showed higher activation in cortical areas when solving the administered task. In particular, the MS participants had higher activation in the left PPC; a brain region whose activity also was correlated to perceived fatigue. It is notable that, according to the theory of central fatigue posed by Chaudhuri and Behan (2000, 2004), central fatigue is defined as a deficit, which is not related to cognitive and motor dysfunction. According to our findings, central fatigue might not be related to per se cognitive dysfunction as the MS participants and controls performed equally well in less complex cognitive tasks. However, fatigue in MS might cause reduced capacity for challenging, complex cognitive tasks. This is an issue that must be addressed in future studies.

In contrast to the findings of hyperactivation in the parietal cortex, during the complex working memory task, MS participants showed less activation in the thalamus and basal ganglia and also in the right DLPFC. In a positron emission tomography (PET) study, Roelcke et al. (1997), found that MS patients with fatigue had decreased glucose metabolism in the frontal cortex and the basal ganglia compared to MS patients without fatigue. They also found that Fatigue Severity Scale (FSS) scores were negatively correlated with regional cerebral glucose metabolism in the right prefrontal cortex (BA 9/10). The authors suggested, in line with the theory by Chaudhuri and Behan (2000, 2004), that demyelination of frontal white matter gives rise to disruption of the cerebral circuits connecting the cortex and basal ganglia, which in turn causes fatigue. That theory is supported by more recent reports on the basal ganglia and cortical atrophy (Calabrese et al. 2010) and reduced white matter integrity in fronto-striatal networks (Pardini et al. 2010) in MS patients with fatigue, as well as decreased creatine (a cellular energy biomarker) levels in the basal ganglia in fatigued HIV-infected individuals (Schifitto et al. 2011).

In the current study, perceived fatigue ratings were positively correlated with activation in the right substantia nigra. MS participants with fatigue also had stronger couplings between the substantia nigra and the thalamus as compared to the control group. According to the theory, GABAergic neurons in the substantia nigra pars reticulata project to the thalamus and thereby inhibit the neural activity of the thalamus, which in turn provides less excitatory output to the cortex (Alexander and Crutcher 1990). In Figure 1, Alexander and Crutcher's model of basal ganglia function is schematically described. According to that theory, there are two parallel pathways within the basal ganglia–thalamocortical circuits having partly opposing effect on the thalamocortical output. The “direct pathway” arises from inhibitory efferents acting on the globus pallidus interna and substantia nigra reticulata. These inhibitory efferents result in less inhibition of the thalamic stage of the circuit. The “indirect pathway” passes through the globus pallidus externa to the subthalamic nucleus. Inhibition of globus pallidus externa results in less inhibition of the subthalamic nucleus, which exerts excitatory signals to the globus pallidus interna and substantia nigra reticulata. Thus the “indirect pathway” results in increased inhibition of the thalamus.

One interpretation of our results might be that a higher activation of the substantia nigra in more fatigued participants results in a greater inhibition of thalamic influences of the cortex, which in turn leads to more fatigue. However, this interpretation remains tentative, especially as we could not discriminate the pars reticulata from the pars compacta of the substantia nigra in the ROI analysis. Another, perhaps somewhat contradictory, finding was that the controls showed more activation of the left substantia nigra compared to MS participants with fatigue whereas more fatigued participants showed more activation of the right substantia nigra. This asymmetry might be caused by the functional variety of different neurons of the substantia nigra. However, the spatial and temporal resolution of current fMRI is not sufficient for such detailed analysis.

Perceived fatigue ratings were also positively correlated with activation of the left PPC. Results from the functional connectivity analysis showed that MS participants had stronger couplings between the PPC and DLPFC compared to controls. We interpret this finding as being in keeping with previous results of studies on the hyperactivation of cortical task-related areas (Lenzi et al. 2008; Genova et al. 2009). Another finding related to the fatigue correlated activity in the parietal cortex was that MS participants exhibited weaker couplings between the left PPC and the right caudate. Thus the MS participants had stronger intracortical couplings (PPC ⇒ DLPFC) and weaker striato-cortical couplings (PPC ⇒ Caudate) involving the parietal cortex. The findings of aberrant couplings of the PPC in MS participants is in agreement with the hypothesis that fatigue may be related to an attention deficit (Calabrese et al. 2010), as the PPC is an important node in the posterior attentional system (Posner 1990). Interestingly, Pellicano et al. (2010)reported that modified FIS scores correlated with the cortical thickness of the parietal lobe.

MS participants had less activation in several areas of the basal ganglia, including the caudate nucleus. In addition, the MS participants had weaker couplings between the parietal lobe and the right caudate compared to controls, as assessed by the functional connectivity analysis. Other evidence of the importance of the caudate in the pathophysiology of fatigue in MS is given by the study by Roelcke et al. (1997). In their PET study they found reduced glucose metabolism in the caudate, especially on the right side. In this context it is interesting to note that lesions to the caudate are related to lack of initiative and poor motivation (Bhatia and Marsden 1994). From our study, we cannot directly conclude if there are disruptions of the striato-cortical pathways or dysfunction of especially the caudate in the striatum that causes weaker functional connectivity in MS patients with fatigue. Nevertheless, these results indicate that the thalamo-striato-cortical network is involved in the pathophysiology of fatigue in MS and perhaps in central fatigue in general.

There are methodological weaknesses in this study regarding the small sample but also the inclusion into the study, giving a selection of persons with MS who also had heat sensitivity. On the other hand heat sensitivity is common in MS; 60–80% are figures mentioned in scientific reports including our own, which means that the sample taken for this study still should be regarded as representative (Flensner et al. 2011). On the other hand, it would of course be interesting to analyze if the magnitude and quality of cognitive dysfunction function in persons with MS differ according to if they are heat sensitive or not. This would be the scope of further study. We suggest that effect of this selection bias (heat sensitivity) on the results of this study may be that the cognitive dysfunction in our population is worse than in an unselected MS population. In addition, pharmaceutical treatment concerning immunomodulating therapy or psychotropic drugs could have influenced the results. The character of this small study, being a pilot study to test the plausibility of central neuronal networks having an impact on cognition, implies that it cannot control for these potential confounders, as such medications are common in an MS population and for ethical reasons hard to stop to improve the study design. Further studies are needed to clarify these issues.

## Conclusions

The aim of the current study was to explore if dysfunction of the thalamo-striato-cortical network could be a factor that explains fatigue in MS. The main findings were that MS participants showed altered brain responses in the thalamo-striato-cortical network during performance of a complex working memory task that challenged fatigue and that brain activation in certain cortical and subcortical areas of the network (the left PPC and the right substantia nigra) was positively correlated to perceived fatigue ratings. Furthermore, MS participants had different functional connectivity between these fatigue-correlated areas and other nodes in the thalamo-striato-cortical network as compared to controls. In particular, MS participants had stronger cortical-to-cortical and subcortical-to-subcortical connections whereas they had weaker cortical-to-subcortical connections. Thus the findings in the present study indicate that the thalamo-striato-cortical network is involved in the pathophysiology of fatigue in MS, and they provide support for the theory of central fatigue in MS. However, due to the limited number of participants and the somewhat heterogeneous sample of MS participants these results have to be regarded as tentative, though they might serve as a basis for future studies.
